# Empowering prevention: uterine cancer awareness and advocacy in the digital age and world of social media

**DOI:** 10.1007/s00404-026-08438-8

**Published:** 2026-05-26

**Authors:** Esra Bilir, Wasim Ahmed, Xezal Derin, Joanna Kacperczyk-Bartnik, Christina Fotopoulou, Sara Nasser

**Affiliations:** 1 Department of Gynecologic Oncology, Koç University School of Medicine, Koç Üniversitesi Rumelifeneri Yolu, 34450 Sarıyer, Istanbul Turkey; 2 Department of Obstetrics and Gynecology, University Medical Center Goettingen, Goettingen, Lower Saxony Germany; 3 Gynecologic Oncology, Pan-Arabian Research Society of Gynecological Oncology, Berlin, Germany; 4 Hull University Business School, Hull, England; 5 Department of Gynecology and Tumor Surgery, Charite Comprehensive Cancer Center, Berlin, Germany; 6 Division Obstetrics and Prenatal Medicine, Goethe University Frankfurt-Main, Frankfurt, Germany; 7 II Department of Obstetrics and Gynaecology, Medical University of Warsaw, Warsaw, Poland; 8 Department of Gynaecological Oncology, Faculty of Medicine, Imperial College London, London, UK; 9 UOC Ginecologia Oncologica, Dipartimento Di Scienze Della Salute Della Donna, del Bambino E Di Sanità Pubblica, Fondazione Policlinico Universitario Agostino Gemelli IRCCS, Rome, Italy

**Keywords:** Endometrial cancer, Uterine Cancers, Social media, Communication, Social Network Analysis, Digital Media

## Abstract

**Purpose:**

We aim to evaluate the reach, engagement, and evolution of the inaugural Uterine Cancer Awareness Month (UCAM) social media campaign (2023–2024) on Twitter/X, focusing on user participation, content trends, and key influencers.

**Methods:**

We conducted a social network analysis of Twitter/X posts using the hashtags #endometrialcancer, #uterinecancer, and #wombcancer over three years (2022–2024). Data were collected and analysed using NodeXL Pro, utilizing the Clauset–Newman–Moore and Harel–Koren Fast Multiscale algorithms for cluster and layout visualization.

**Results:**

In 2022, 343 users generated 557 interactions, emphasizing health disparities and symptom awareness. The engagement peaked in 2023 with 302 users and 731 interactions, driven by strategic hashtag use and awareness efforts. A decline was noted in 2024 with 237 users and 484 interactions. Word pair analysis showed evolving themes, from general symptom awareness in 2022 to targeted messaging around advanced cases and recurrence in 2024.

**Conclusion:**

The UCAM-social media campaign showed promising initial growth but experienced a decline in engagement by 2024, highlighting the need for sustained and diversified strategies. Our study, which assessed the campaign’s impact over a period exceeding one year, a rarity in the current literature, highlights critical insights for future initiatives.

**Supplementary Information:**

The online version contains supplementary material available at 10.1007/s00404-026-08438-8.

## What does this study add to the clinical work

**Table:** 

Our study provides longitudinal evidence showing that social media awareness campaigns in uterine cancer shape the type and depth of patient knowledge, and generate actionable insights to guide clinicians and institutions in optimizing digital strategies that promote early diagnosis and patient-centered care in uterine cancer.	

## Introduction

The global incidence and mortality of uterine cancer in 2022 were 420,242 and 97,704 cases, respectively, according to GLOBOCAN estimates based on data from 185 countries covering 36 types of cancer [1]. In regions and countries where cervical cancer has a high incidence and mortality, the incidence and mortality of endometrial cancer are comparatively low and are positively correlated with the Human Development Index [2]. Population-based registry data in Sweden indicate the incidence of endometrial cancer more than doubled between 1960 and 2014 [3]. The crude incidence rate increased from 16.2 per 100,000 women in 1960 to 28.6 per 100,000 women in 2014 [3].

The established association between obesity, its related comorbidities, and the increased risk of uterine cancer underscores the importance of raising awareness about this malignancy. Given these, uterine cancer, such as cervical cancer, could be viewed as a potentially preventable malignancy. There is a growing recognition of the need for a more comprehensive and preventive approach to uterine cancer and uterine pre-malignancies and their associated risk factors. Due to the increasing incidence of endometrial cancer among younger populations, fertility-sparing treatments and strategies to preserve fertility become crucial components of patient care with minimally invasive techniques [4, 5].

In the post-COVID era, the influence and reach of social media in medicine become increasingly significant for both patients and healthcare professionals. The introduction of gynaecologic oncology-specific hashtags (#goASCO20 and #goASCO21), distinct from the official conference hashtags, significantly enhanced discussions relevant to gynaecologic oncologists during virtual meetings [6]. In raising awareness for gynaecologic cancers, Twitter, also known as X, campaigns were reported to be effective when centered around a single, easy-to-spell hashtag and coordinated with influential accounts through timed tweets during World Gynecologic Oncology Day, an awareness social media campaign run by The European Network of Gynaecological Cancer Advocacy Groups (ENGAGe) [7]. Twitter/X serves as a key platform for sharing scientific information among gynecologic oncologists through social media ambassadors and collaborations with networks, such as OncoAlert [8]. Implementing official ambassador programs and partnering with influential accounts significantly boost engagement and visibility for congress-related content on Twitter/X [8]. Social media, particularly Twitter/X, increases the visibility of scientific journals and is increasingly used by surgical societies to promote meetings and disseminate research. A review identified that journal articles including gynaecologic oncology studies, since the formation of the Social Media Committee, exhibit the highest engagement on Twitter/X [9].

Similarly, Twitter/X, and Instagram were reported to be effective social media tools for various gynaecologic cancers, including cervical cancer vaccine promotion and elimination efforts [10–16]. Despite controversies, such as the temporary blocking of the hashtag #VaginalCancer on Instagram, these platforms remain essential for cancer education and advocacy, highlighting the need for continuous scrutiny and advocacy to ensure unrestricted access to vital cancer-related information [17].

On April 28, 2023, the International Gynecologic Cancer Society (IGCS) Advocacy Network, in collaboration with the European Network of Gynaecological Cancer Advocacy Groups (ENGAGe) and other partners, designated June as Uterine Cancer Awareness Month (UCAM). The campaign was promoted across various social media platforms, including Twitter/X, Instagram, and Facebook, using the hashtag #uterinecancer. Our study evaluates the reach and impact of this initiative on Twitter/X.

## Methodology

We conducted a comprehensive search on X (formerly Twitter) for tweets containing the hashtags #endometrialcancer, #uterinecancer, or #wombcancer over a three-year span: 2022(the year preceding the campaign), 2023(the campaign’s inaugural year), and 2024(the campaign’s second year). The selected hashtags, #endometrialcancer and #uterinecancer, were chosen in accordance with the Joint Statement: Gynecology Social Media Ontologies where IGCS is one of the societies [18]. To ensure coverage of British English terminology, the hashtag #wombcancer was also included.

Data collection and analysis were carried out using NodeXL Pro. Our dataset includes all users who posted with these hashtags, and also those who were mentioned, replied to, retweeted, or quoted in relation to these hashtags.

We analyzed the data in NodeXL Pro drawing upon social network analysis [19]. We conducted a network analysis where vertices were clustered by the Clauset-Newman-Moore cluster algorithm [20]. The graphs were laid out using the Harel-Koren Fast Multiscale layout algorithm [21]. There is an edge for each "replies-to" relationship in a post, an edge for each "mentions" relationship in a post, an edge for each "repost" relationship in a post, an edge for each "quote" relationship in a post, an edge for each "mention in repost" relationship in a post, an edge for each "mention in reply-to" relationship in a post, an edge for each "mention in quote" relationship in a post, an edge for each "mention in quote reply-to" relationship in a post, and a self-loop edge for each post that is not from above.

Our analysis included the total unique Twitter/X accounts as well as the total interactions, encompassing posts and tweets. We reported the top 10 Twitter/X accounts, listing each by rank, vertex, betweenness centrality, name, role, and classification. The term "rank" refers to the position of an account based on its overall significance or influence within a network. A "vertex" represents a specific Twitter/X account within the network graph, while "betweenness centrality" measures how frequently an account serves as a bridge along the shortest path between two other accounts, thereby indicating its influence over the flow of information. The "name" denotes the actual username or handle of the Twitter/X account. We define "role" as the function or contribution of the corresponding account within the network, which may include classifications such as gynaecologic oncologist, medical society, influencer, patient, scientific society, hospital, and pharmaceutical company.

Our objective is to refine the classification of Twitter/X accounts by grouping them according to specific areas of interest. We categorized these accounts into four distinct groups for our analysis: scientific community, which encompasses journals, societies, and medical centers; physician, referring to users who claim to hold a medical degree; patient advocacy, representing groups that support and advocate for patient rights and needs; and company, which includes entities with profit-oriented goals. The information was obtained from user profile descriptions. We identified the top 10 co-occurring word pairs, presenting each pair (word 1 and word 2) alongside its frequency count. 

In accordance with the journal’s guidelines, we will provide our data to a team selected by the Editorial Team for independent analysis for the purposes of additional data analysis or for the reproducibility of this study in other centers if such is requested.

## Results

**Fig. 1:**
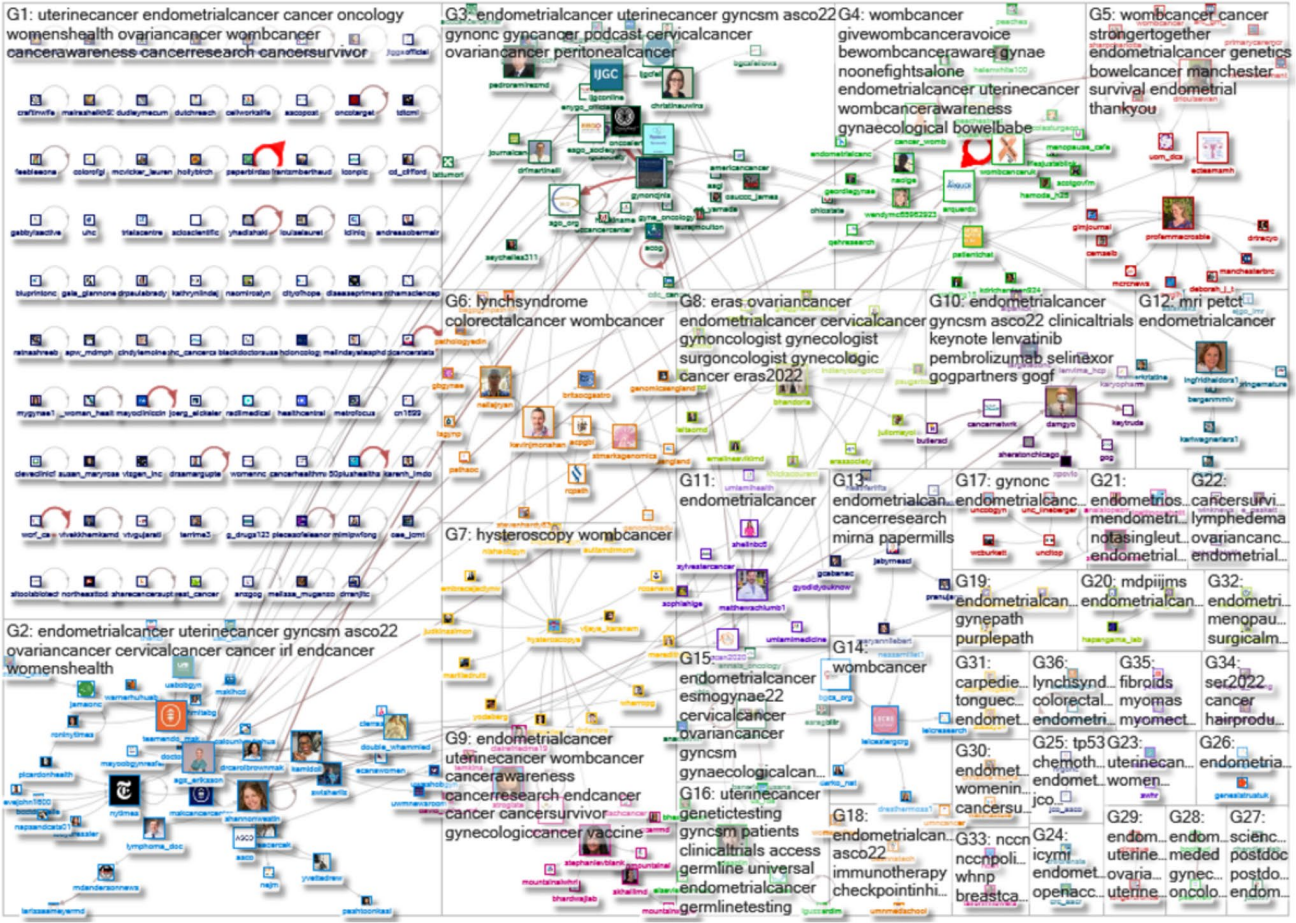
Social Network Analysis in 2022

We showed a network of 343 Twitter/X users and 557 interactions related to discussions on uterine cancer (Fig. 1). Interaction patterns were used to classify users into groups. In the network figure, accounts are represented with larger node sizes, indicating a higher level of influence. The visualization highlights the formation of several distinct communities, each focused on different facets of uterine cancer. Despite some degree of interconnectivity among these communities, most clusters remain relatively small. Table 1 presents a ranking of the top 10 influencers from 2022 who were most active in these discussions.

**Table 1:** Top 10 X Influencers in 2022

Rank	Vertex	Betweenness Centrality	Name	Role	Classification
1	agz_eriksson	21,017.414	Ane Gerda Z Eriksson MD PhD	Gynecological Oncologist	Physician
2	gynoncjnls	11,005.882	@GynOncJnls	Medical Journal	Scientific community
3	ecteamsmh	9727.600	Team Womb	Endometrial Cancer Research Team	Scientific community
4	profemmacrosbie	8439.800	Emma Crosbie	Gynecological Oncologist	Physician
5	ijgconline	7427.186	IJGC	Medical Journal	Scientific community
6	shannonwestin	6603.898	Shannon Westin, MD, MPH, FASCO	Gynecological Oncologist	Physician
7	neilajryan	5628.000	neil ryan	Gynecological Oncologist	Physician
8	gyncsm	5265.770	GYN Cancer | #GYNCSM	Advocacy and Support Community for gynecological cancer	Patient advocacy
9	bhandoria	4597.000	Geetu Bhandoria	Gynecological Oncologist	Physician
10	sgo_org	3920.129	SGO	Medical Society	Scientific community

In June 2022, we observed the following word pair frequencies: Black Women (25 occurrences), Endometrial Cancer (24 occurrences), Uterine Cancer (20 occurrences), Know Signs (16 occurrences), Need Know (15 occurrences), Symptom Cards (14 occurrences), and #WombCancer (14 occurrences) (Supplementary Table S1).

**Fig. 2:**
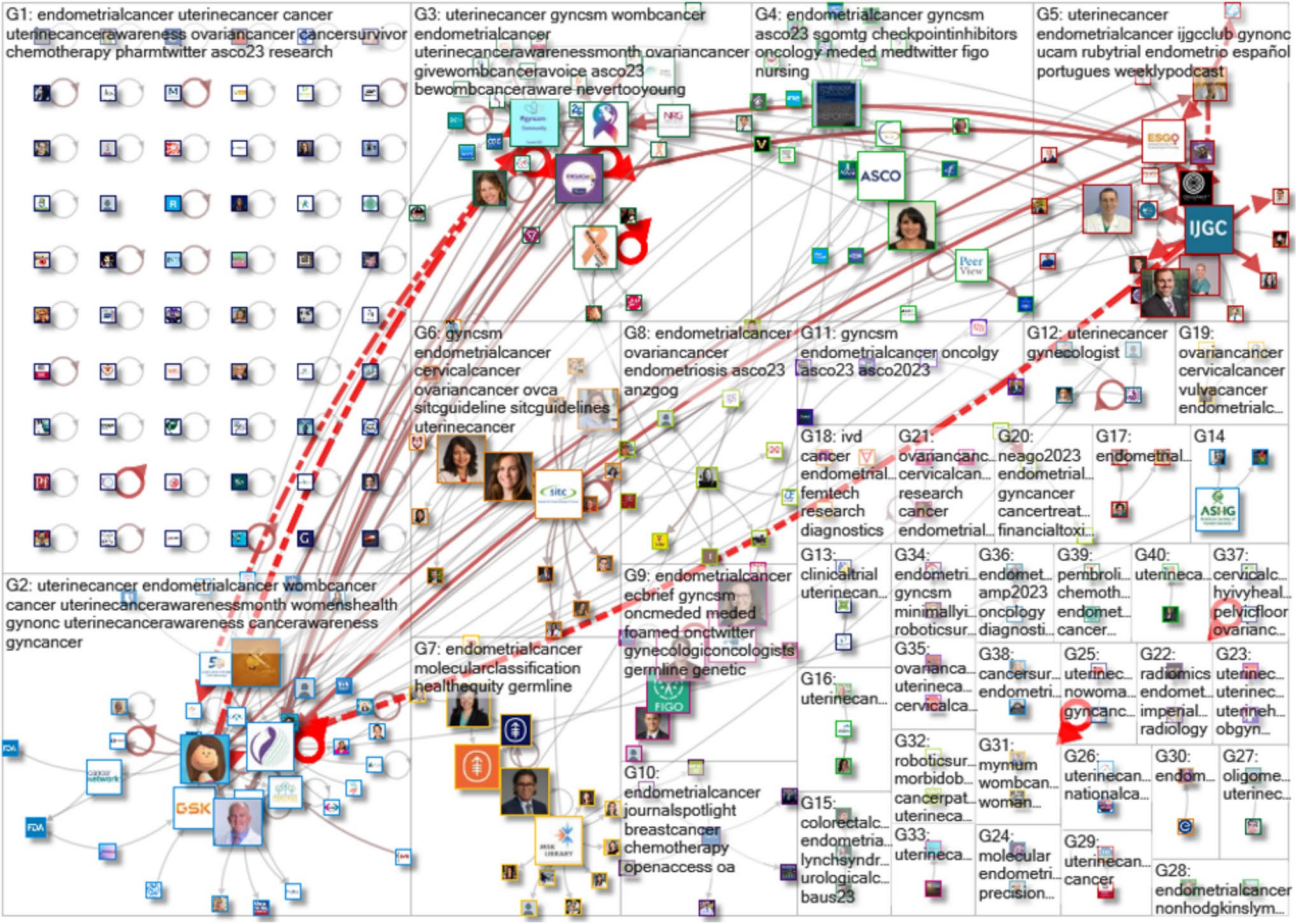
Social Network Analysis in 2023

We illustrated a network comprising 302 Twitter/X users and 731 interactions among them in Fig. 2. The tweets within this network were collected over a period spanning 291 days, 20 h, and 31 min, from Friday, June 23, 2023, at 18:15 UTC to Wednesday, April 10, 2024, at 14:46 UTC. Table 2 provides a detailed analysis of co-words, highlighting the frequency of word pairs associated with uterine cancer awareness. This analysis offers insights into the predominant topics and themes within awareness campaigns and related literature.

**Table 2:** Top 10 X Influencers in 2023

Rank	Vertex	Betweenness Centrality	Name	Role	Classification
1	strogista	8106.521	Elizabeth Hornsey	Patient Advocate, Cancer Survivor	Patient advocacy
2	igcsociety	6848.616	IGCS	Medical Society	Scientific community
3	gynoncjnls	5741.266	@GynOncJnls	Medical Journal	Scientific community
4	gyncsm	4973.294	GYN Cancer | #GYNCSM	Advocacy and Support Community	Physician
5	sitcancer	4548.496	Society for Immunotherapy of Cancer	Medical Society	Scientific community
6	ijgconline	2710.549	IJGC	Medical Society	Scientific community
7	msklibrary	2512.621	MSK Library	Medical Library	Scientific community
8	lamcalarnenmd	2381.198	Lindsey McAlarnen, MD, FACOG	Gynecological Oncologist	Physician
9	drfmartinelli	1950.448	Fabio Martinelli	Gynecological Oncologist	Physician
10	riosdoriamd	1808.437	Eric Rios-Doria, MD	Gynecologic Oncoloy Fellow	Physician

In June 2023, we recorded the following word pair frequencies: Awareness Month (60 occurrences), Endometrial Cancer (53 occurrences), Uterine Cancer (51 occurrences), #EndometrialCancer (42 occurrences), #EndometrialCancer #WombCancer(41 occurrences), #UterineCancer Awareness (32 occurrences), Cancer Awareness(25 occurrences), Learn More (23 occurrences), Signs Symptoms (22 occurrences), and #Gyncsm #UterineCancer (21 occurrences) (Supplementary Table S2).

**Fig. 3:**
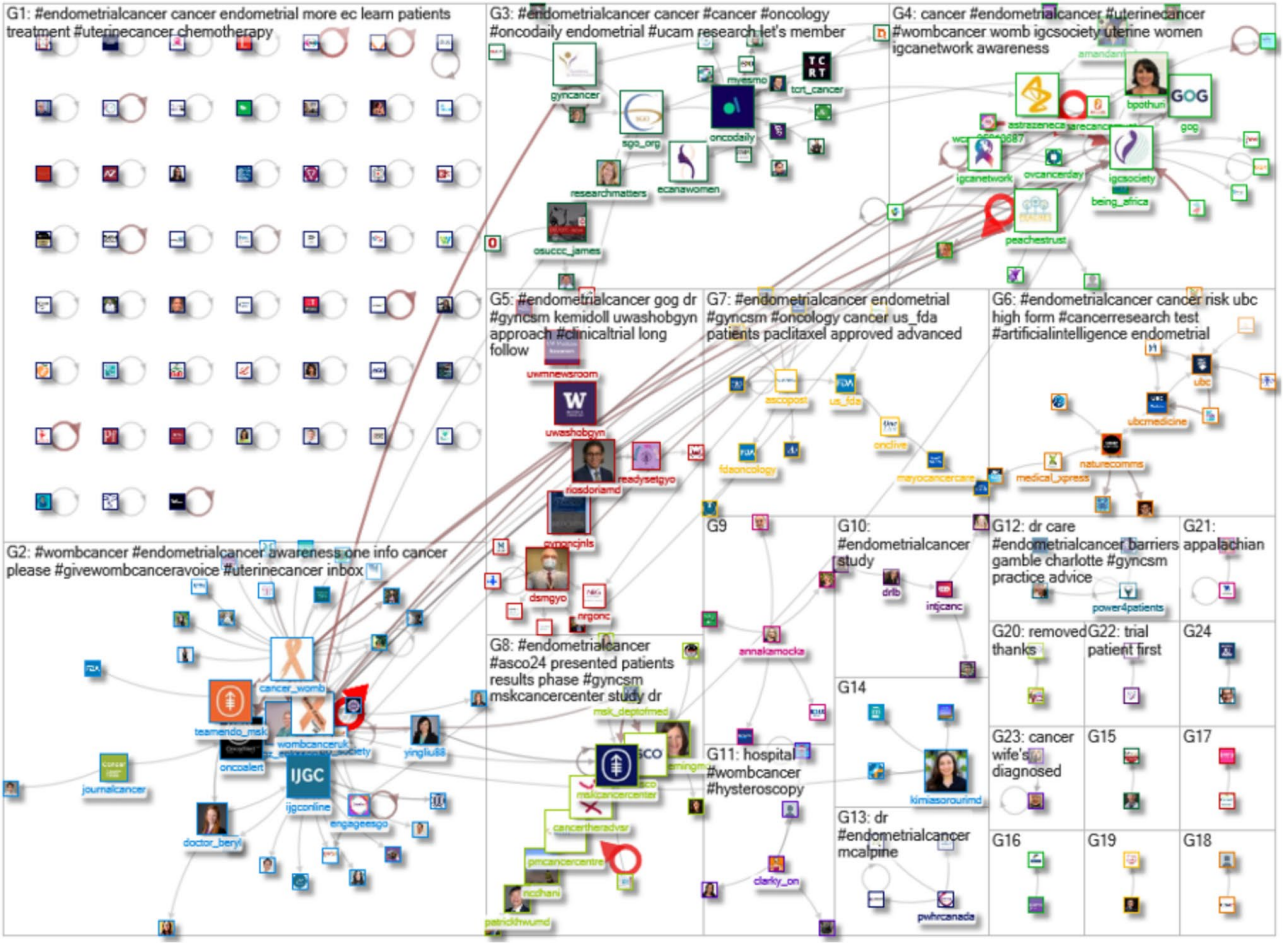
Social Network Analysis in 2024

We visualized a network of 237 Twitter/X users and 484 interactions, as shown in Fig. 3. This network includes users who recently tweeted with the hashtags "#endometrialcancer," "#uterinecancer," or "#wombcancer," as well as users who were mentioned, replied to, retweeted, or quoted in relation to these tweets. The dataset was limited to 20,000 tweets collected between June 1, 2024, at 00:00 UTC, and June 30, 2024, at 23:55 UTC. Data collection occurred on October 28, 2024, at 12:50 UTC. Table 3 presents the Top 10 Influencers on X for 2024, while Supplementary Table S3 provides a detailed co-word analysis, identifying common word pairs linked to uterine cancer awareness. This analysis sheds light on key topics and themes within awareness campaigns and relevant discussions.

**Table 3:** Top 10 X Influencers in 2024

Rank	Vertex	Betweenness Centrality	Name	Role	Classification
1	igcsociety	4111.044	IGCS	Medical Society	Scientific community
2	cancer_womb	3364.104	Womb Cancer Awareness	Non- profit charity organization for Endometrial Cancer	Patient advocacy
3	ijgconline	3225.575	IJGC	Medical Journal	Scientific community
4	oncodaily	2595.067	OncoDaily	Media & News Company	Scientific community
5	wombcanceruk	2412.809	Womb Cancer Support UK	Support and Awareness Organization	Patient advocacy
6	bpothuri	2103.448	Bhavana Pothuri	Gynecologic Oncologist and Patient Advocate	Physician
7	mskcancercenter	1696.881	Memorial Sloan Kettering Cancer Center	Cancer Center	Scientific community
8	riosdoriamd	1530.294	Eric Rios-Doria, MD	Gynecologic Oncology Fellow	Physician
9	astrazeneca	1490.663	AstraZeneca	Pharmaceutical Company	Company
10	teamendo_msk	1450.237	MSKCC Team Uterus	Endometrial Cancer Service	Scientific community

In our findings from the 2024 data, the most frequent word pair was ‘Endometrial Cancer,’ ranking first with 51 occurrences. The hashtag combination ‘#UterineCancer #EndometrialCancer’ ranked second with 40 occurrences, followed by ‘Uterine Cancer’ in third place with 32 occurrences. Another instance of ‘Endometrial Cancer’ ranked fourth with 26 occurrences, while ‘#WombCancer #UterineCancer’ and ‘#UterineCancer #EndometrialCancer’ ranked fifth and sixth with 23 and 22 occurrences, respectively. The term ‘Womb Cancer’ appeared twice, ranking seventh with 21 occurrences and eighth with 20. Rounding out the list, both ‘Learn More’ and ‘Advanced Recurrent’ shared the ninth rank, each with 17 occurrences.

Between 2022 and 2023, there was a notable decline in the number of users. The user count decreased from 343 in 2022 to 302 in 2023, which represents a 12.0% decrease. This downward trend continued into 2024, where the number of users fell further to 237, marking a 21.5% decrease compared to the previous year. This consecutive reduction indicates a significant drop in user engagement over the two-year period. In contrast, tweet activity showed a different pattern. From 2022 to 2023, the number of tweets increased from 557 to 731, reflecting a 31.2% increase. This rise in tweet volume suggests that, although fewer users were active, those remaining were more engaged or posting more frequently. However, in 2024, tweet activity saw a decline, dropping from 731 in 2023 to 484 tweets in 2024, a 33.8% decrease. This shift suggests both user numbers and tweet activity began to wane significantly by 2024, indicating a possible overall reduction in platform engagement.

In 2022, the *physician* category was the most prominent, making up 50% (*n* = 5) of the users, followed by the *scientific community* at 40% (*n* = 4) and *patient advocacy* at 10% (*n* = 1). In 2023, there was a shift, with the *scientific community* becoming the largest category at 50% (*n* = 5), *physicians* decreasing to 40% (*n* = 4), and *patient advocacy* remaining at 10% (*n* = 1). In 2024, the *scientific community* maintained its lead at 50% (*n* = 5), while *patient advocacy* increased to 20% (*n* = 2), *physicians* decreased to 20% (*n* = 2), and *companies* appeared as a new category at 10% (*n* = 1).

## Discussion

Our study showed the Twitter/X campaign on uterine cancer awareness evolved from 2022 to 2024, starting with efforts among 343 users and 557 interactions. Early discussions were centered around “Black women”, emphasizing health disparities and a need for awareness on symptoms and signs of uterine cancer. The focus was on practical educational tools, such as symptom cards, and terms, such as "Know Signs." By 2023, engagement increased, and the campaign gained traction with 302 users and 731 interactions, marked by a strategic use of hashtags (#UterineCancer, #EndometrialCancer) and phrases, such as"Awareness Month" and "Learn More." The hashtag combinations indicated a concerted effort to unify awareness under a shared digital community, emphasizing early detection and public education on uterine cancer. Interestingly, we did not find word pairings on “risk factors” or “prevention” for endometrial cancer.

In 2024, participation narrowed to 237 users with 484 interactions, but core themes remained strong with a refined focus on impactful hashtags and specific terms, such as "Advanced Recurrent," pointing to discussions around complex cases and recurrences. Potential external contributors to the decline from 2023 to 2024 may include modifications to the Twitter/X platform (e.g., algorithmic changes, policy revisions, or user migration), concurrent awareness campaigns, or broader societal events that could have influenced user engagement.

Overall, the campaign matured from a broad, community-specific initiative in 2022 to a peak of coordinated social media strategies in 2023, consolidating in 2024 with targeted messaging to sustain engagement among core audiences, particularly on uterine and endometrial cancers, emphasizing both general awareness and nuanced aspects, such as recurrence. Even between 2022 and 2024 there was little visible focus on prevention and risk factors within the social network analysis. This reflects the persistent lack of prevention strategies when raising awareness on certain cancers. Obesity is a significant risk factor for endometrial cancer. Our social network analysis over the last 3 years reflects that cancer should not be stigmatized. We note an insufficient emphasis on the fact that many cancers are preventable.

There is a shift to educate on prevention and empower women to reduce their risk factors themselves. This is evidenced from 2022 to 2024, representation within the community shifted as physicians declined from 50 to 20%, the scientific community maintained a leading 50% share, patient advocacy grew from 10 to 20%, and companies emerged at 10%, illustrating an evolving presence of advocacy and profit-oriented entities. Our findings demonstrated a lack of presence of ENGAGe among the top influencers, with individual accounts being more prominent. This observation aligns with the previous social media ambassador program, which revealed to amplify the dissemination of gynaecologic oncology–related information across social media [8].

The introduction of the new hashtags #goASCO20 in 2020 and #goASCO21 in 2021 at the American Society of Clinical Oncology (ASCO) virtual conferences in 2020 and 2021 demonstrated a significant increase in engagement, with tweets using the #goASCO21 hashtag receiving 360 interactions compared to 75 for #goASCO20 (z value of 16.63 and *p*-value < 0.001), indicating a notable rise in engagement in the subsequent year [6]. However, our analysis indicates the UCAM campaign on Twitter/X did not show the expected increase in the second year (2024) as compared to the first year (2023).One possible reason for this trend might be the introduction of a new hashtag during the ASCO virtual conferences held during the COVID pandemic [6], when the society likely had more time and interest in engaging with social media. This was supported with the following data during the COVID-19 pandemic, social media use surged globally, with U.S. users averaging 65 min daily in 2020, up from 54 and 56 min in the previous years [22].

The Society of Gynecologic Oncology (SGO) launched the #SGOatASCO campaign during the first virtual ASCO Congress, where it generated 48 original tweets [23]. In contrast, the #goASCO20 campaign produced 165 original tweets [23]. Although both campaigns were introduced in 2020, there is more than a threefold difference in the number of tweets [23]. This discrepancy highlights the potential fluctuations in social media engagement. Our results indicate the main campaign leader, the IGCS, was not among the top 10 users in 2022 but ranked second in 2023 and first in 2024. However, the partner societies did not appear in the top 10 during any of the years analysed in our study. This underscored the critical role played by the stakeholders behind the social media campaign.

In the literature, several studies highlight the importance of using specific hashtags in social media for cancer awareness and prevention campaigns, particularly for gynaecological and breast cancers, with analyses often focusing on individual years in Twitter/X [7, 24] Another Twitter/X study examining cancer-related 7,753 tweets in Japan for 2022, focusing on breast, lung, and colon cancer, categorized the 4976 accounts into seven types (survivor, patient’s family, healthcare provider, public organization, private organization, news, and other) [25]. Their findings revealed breast cancer-related tweets were the most frequent (62.8%), primarily from survivor accounts, while lung cancer tweets were often from Patient’s family accounts, highlighting the importance of understanding the characteristics of information sources on social media [25]. The analysis based on a single year cannot be generalized to subsequent years. Demographic characteristics, such as age and location, play a crucial role in influencing social media usage and the choice of social media platforms, as not all channels are accessible in every country.

Our study has several limitations. First of all, we focused on a single social media platform, whereas analysing multiple platforms could offer a broader user perspective. Moreover, our analysis was restricted to English language content and generic hashtags, potentially overlooking more specific and inclusive hashtags. Collectively, these factors could limit the generalizability of our findings across different languages and cultural contexts. Our methodology was limited to hashtag searches, which may have excluded relevant discussions of uterine cancer that did not use hashtags. One of our strengths is the assessment of the impact of the social media campaign over multiple years, unlike many single-year analyses.

The observed shift towards more organized advocacy and strategic awareness in uterine cancer discussions underscores the importance of integrating targeted awareness campaigns into practice. Given that uterine cancer is often preventable when risk factors and comorbidities are properly managed, efforts should focus on enhancing awareness of these risk factors and preventive measures. Our analysis shows there is a movement towards more patient empowerment to take responsibility for preventing and reducing risk factors as well as seeking treatment.

Future research should explore the application of artificial intelligence-driven tools to analyse language patterns and outreach effectiveness, including click rates, to refine communication strategies and reach broader audiences. Developing region-specific solutions tailored to local needs and integrating these approaches with patient advocacy groups could further improve outreach and impact. This aligns with the recommendations and efforts of the international societies and patient advocacy networks of the International Gynecological Cancer Advocacy Network (IGCAN), the Pan-Arabian Research Society of Gynecological Oncology (PARSGO) and ENGAGe [7, 26, 27]. By combining these strategies, we can advance both preventive efforts and public awareness in a more targeted and effective manner.

Recommendations for future campaigns include the development of a unified communication strategy across various stakeholders to ensure consistent messaging, the expansion of outreach efforts to additional social media platforms beyond Twitter/X to broaden audience engagement, and a stronger emphasis on prevention education to increase public awareness and encourage proactive health behaviours.

## Conclusion

Our analysis involving the social media campaign from Twitter/X on UCAM evolved from small, targeted communities in 2022 to a robust, widespread campaign in 2023. The UCAM campaign’s reach was maximized through coordinated hashtags and clear calls to action. In 2024, despite a drop in engagement and user numbers, the UCAM Twitter/X campaign consolidated its core mission, focusing on targeted educational themes around uterine cancer by not placing sufficient emphasis on important topics such as “risk factor”’ and “prevention”. This progression highlights a maturation of social media strategies in health awareness, evolving from broad awareness-building to a more concentrated focus on impactful keywords and refined educational content.

## Supplementary Information

Below is the link to the electronic supplementary material.

Supplementary file1 (DOCX 11 KB) Table S1: Top co-words in 2022

Supplementary file2 (DOCX 11 KB) Table S2: Top co-words in 2023

Supplementary file3 (DOCX 10 KB) Table S3: Top co-words in 2024

## Data Availability

No datasets were generated or analysed during the current study.
