# Supplementary material for: Empowering prevention: uterine cancer awareness and advocacy in the digital age and world of social media
Source: Arch Gynecol Obstet. 2026 May 26;313(1):231. doi: 10.1007/s00404-026-08438-8 (PMC13384964; doi:10.1007/s00404-026-08438-8)
Supplement: Supplementary file 1 — Supplementary file1 (DOCX 11 KB) Table S1: Top co-words in 2022 [file 404_2026_8438_MOESM1_ESM.docx]

**Table 1**: Top co-words in 2022

| **Word 1** | **Word 2** | **Count** |
| --- | --- | --- |
| 1. black | women | 25 |
| 1. endometrial | cancer | 24 |
| 1. uterine | cancer | 20 |
| 1. know | signs | 16 |
| 1. need | know | 15 |
| 1. symptom | cards | 14 |
| 1. batch | small | 14 |
| 1. give | anyone | 14 |
| 1. delivered | ideal | 14 |
| 1. small | #wombcancer | 14 |
