# Supplementary material for: Empowering prevention: uterine cancer awareness and advocacy in the digital age and world of social media
Source: Arch Gynecol Obstet. 2026 May 26;313(1):231. doi: 10.1007/s00404-026-08438-8 (PMC13384964; doi:10.1007/s00404-026-08438-8)
Supplement: Supplementary file 2 — Supplementary file2 (DOCX 11 KB) Table S2: Top co-words in 2023 [file 404_2026_8438_MOESM2_ESM.docx]

**Table 2:** Top co-words in 2023

| **Word 1** | **Word 2** | **Count** |
| --- | --- | --- |
| 1. awareness | month | 60 |
| 1. endometrial | cancer | 53 |
| 1. uterine | cancer | 51 |
| 1. #uterinecancer | #endometrialcancer | 42 |
| 1. #endometrialcancer | #wombcancer | 41 |
| 1. #uterinecancer | awareness | 32 |
| 1. cancer | awareness | 25 |
| 1. learn | more | 23 |
| 1. signs | symptoms | 22 |
| 1. #gyncsm | #uterinecancer | 21 |
