# Supplementary material for: Empowering prevention: uterine cancer awareness and advocacy in the digital age and world of social media
Source: Arch Gynecol Obstet. 2026 May 26;313(1):231. doi: 10.1007/s00404-026-08438-8 (PMC13384964; doi:10.1007/s00404-026-08438-8)
Supplement: Supplementary file 3 — Supplementary file3 (DOCX 10 KB) Table S3: Top co-words in 2024 [file 404_2026_8438_MOESM3_ESM.docx]

**Table 3:** Top co-words in 2024

| **Word 1** | **Word 2** | **Count** |
| --- | --- | --- |
| 1. endometrial | cancer | 51 |
| 1. #uterinecancer | #endometrialcancer | 40 |
| 1. uterine | cancer | 32 |
| 1. endometrial | cancer | 26 |
| 1. #wombcancer | #uterinecancer | 23 |
| 1. #uterinecancer | #endometrialcancer | 22 |
| 1. womb | cancer | 21 |
| 1. womb | cancer | 20 |
| 1. learn | more | 17 |
| 1. advanced | recurrent | 17 |
